# Cold atmospheric plasma generated reactive species aided inhibitory effects on human melanoma cells: an *in vitro* and *in silico* study

**DOI:** 10.1038/s41598-020-60356-0

**Published:** 2020-02-25

**Authors:** Dharmendra Kumar Yadav, Manish Adhikari, Surendra Kumar, Bhagirath Ghimire, Ihn Han, Mi-Hyun Kim, Eun-Ha Choi

**Affiliations:** 1 Gachon Institute of Pharmaceutical Science & Department of Pharmacy, College of Pharmacy, Gachon University, Incheon, Republic of Korea; 2 Plasma Bioscience Research Center, Applied Plasma Medicine Center, Department of Electrical & Biological Physics, Kwangwoon University, Seoul, Republic of Korea

**Keywords:** Basal cell carcinoma, Apoptosis, Cheminformatics, High-throughput screening

## Abstract

Malignant melanoma is considered to be a heterogeneous disease that arises from altered genes and transformed melanocytes. In this study, special softjet cold atmospheric plasma was used to treat three different human melanoma cells using air and N_2_ gases to check the anti-melanoma activity. The physical effects by plasma revealed an increase in the temperature with the gradual reduction in pH at 60 sec, 180 sec and 300 sec air and N_2_ plasma treatment. Cellular toxicity revealed a decreased in cell survival (~50% cell survival using air gas and <~60% cell survival using N_2_ gas at 60 sec plasma treatment in G-361 cells). Gene analysis by q-PCR revealed that 3 min and 5 min air and N_2_ plasma treatment activated apoptotic pathways by triggering apoptotic genes in all three melanoma cell lines. The apoptosis was confirmed by DAPI staining and its related pathways were further explored according to protein-protein docking, and their probable activation mechanism was revealed. The pathways highlighted that activation of apoptosis which leads to cellular cascades and hence stimulation ASK1 (docking method) revealed that softjet plasma can be an effective modality for human melanoma treatment.

## Introduction

Malignant melanoma considered as one type of cancer that primarily originates by a mutation in skin pigment making cells known as melanocytes^1–5^. Melanoma has the highest mortality rate worldwide and remains one of the most prominent types of skin cancer^6^. It is considered to be a heterogeneous disease with complex cellular mechanisms owing to specific genetic alterations that occur within several functionally related molecular pathways^7^. Methods for controlling cancer mostly depend on anticancer agents, which are limited and have after-effects. Thus, there must be an urgent need to adopt new technology for melanoma treatment. Soft jet cold atmospheric plasma (CAP) is a recent technological innovation that has opened a new research perspective to the melanoma treatment and other medical applications^8^, such as treating skin diseases^9–11^, dental problems^12,13^, and wounds^14^. The softjet plasma device uses high voltage and current to discharge plasma by using different gas or gaseous mixtures. The discharged CAP contains ionized gas molecules, electrons, excited atoms, ultraviolet (UV) radiations, electromagnetic fields, etc. The softjet plasma device induced reactive atoms to combine with each other and other molecules to form reactive oxygen and nitrogen species (RONS)^15–17^. These characteristics of soft jet cold atmospheric plasma have encouraged its therapeutic application for melanoma treatment.

Here, we examined the effect of soft jet CAP using air and N_2_ gases on normal human dermal fibroblast (nHDF) and different human melanoma cell lines: G-361, SK-MEL-31, and WM-266-4. CAP is nowadays proposed to be used in cancer treatment, but very few studies and evidence on the effect of softjet CAP on melanoma are available so far. The heating effect and increase in pH are the most common issues with traditional cold plasma technology and can be controlled with a softjet device.

The focus of this study was to estimate the anti-melanoma activity of softjet CAP by using air and N_2_ gases and an evaluation of the plausible mechanism to targeted melanoma treatment. We hypothesize that plasma exposure from different gases individually induces cancer cell death with minimal effects on normal adjacent cells. The results demonstrated the downregulation of metabolic viability with the simultaneous upregulation of apoptosis and its related genes. This new approach toward abrogating the apoptotic pathway using softjet CAP may provide a different strategy for melanoma treatment. We further explored the role of selected reactive oxygen and nitrogen species in modulating the apoptotic pathway through a protein-protein docking simulation. The *in vitro* results showed that soft jet CAP from the air and N_2_ gas can plausibly be used to inhibit melanoma progression.

## Materials and Methods

RPMI-1640, MEM, phosphate-buffered saline, RIPA buffer and the penicillin-streptomycin antibody cocktail solution were obtained from Welgene, Korea. FBS and trypsin were procured from Hyclone (GE Healthcare Life Sciences), and the antibody cocktail used in this study (penicillin and streptomycin) was procured from Gibco, Korea. Nitric oxide and peroxide determination kit were procured from Bioassay Systems, USA. FGM-2, bulletkit was obtained from Lonza, NJ, USA. RNAiso plus (Trizol) was purchased from Takara, Japan. Glass bottom cell culture dishes were purchased from NEST, New Jersey, USA. Nitrocellulose membrane, Isopropanol and Triton X-100 were purchased from Thermofisher, Korea. Diethyl pyrocarbonate (DEPC) and 4% Paraformaldehyde were obtained from Biosesang, Korea. The nucleus staining DAPI dye was procured from Sigma Aldrich, Korea. Anti-rabbit monoclonal cleaved caspase-3 primary antibody was purchased from cell signalling technologies, USA and anti-mouse- GAPDH antibody was procured from Bio-Rad, USA. The ReverTra Ace^®^ qPCR RT Master Mix cDNA synthesis kit was purchased from Toyobo, Japan. The SYBR Green Master mix was procured from Bio-Rad, Korea. All the primers for real-time polymerase chain reaction (q-PCR) analysis were obtained from Searchbio, Korea.

### Device characterization

#### Electrical characteristics

**Figure 1:**
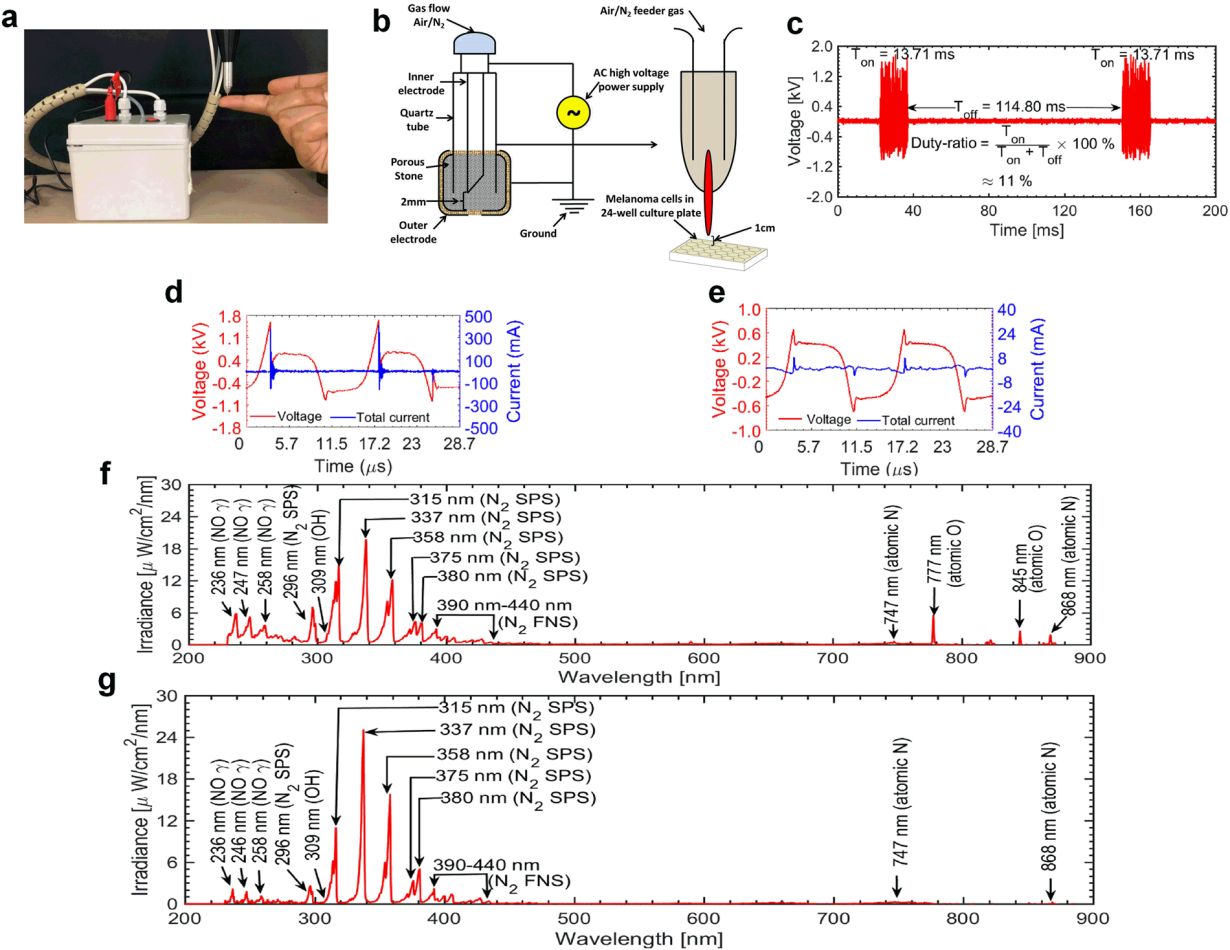
Illustration of softjet plasma jet device with different gas systems. **(a)** Device setup for the experiment; **(b)** Schematic representation of plasma jet on melanoma cells; **(c)** Waveforms showing the on-time and off-time of the plasma jet. Current-voltage waveforms of the discharge during plasma on time for plasma jet operated with **(d)** air gas **(e)** nitrogen gas; Optical characteristics of the plasma jet operated with **(f)** air gas; **(g)** nitrogen gas.

The device used for the experimentation and its diagrammatic illustration was mentioned (Fig. 1a,b). The electrical characteristics (recorded with Lecroy wavesurfer 434, 350 MHZ oscilloscope using Tektronix P6015A high voltage probe and Tektronix P6022 current probe) for the soft plasma jet operated with air and nitrogen gas are shown in Fig. 1d,e. The flow rate of both gases was fixed at 1.5 liters per minute. Plasma was generated in dimming mode by using a DC to AC inverter whose operational time (T_on_) and shutting time (T_off_) were 13.71 ms and 114.80 ms (Fig. 1c). The duty percentage was ~11%. A long shutting of time (~90% of the total duty cycle) was essential in order to prevent heating of the plasma jet and enable longer operational duration. The current and voltage waveforms recorded during the Ton period of the circuit are shown in Fig. 1d,e for air and nitrogen gas respectively. For both air and nitrogen gas, the voltage waveforms appear distorted during each positive and negative half cycle of the applied voltage. The frequency of the source is ~70 kHz. The dissipated power (P) during the discharge was estimated by integrating the voltage (V(t)) and current (I(t)) signals recorded at each time points (t) over one time period (T) of the duty cycle as Kim *et al*. 2019,

$$P=duty-ratio\times {\int }_{0}^{T}I(t)V(t)dt$$

A comparison among the averaged values of applied voltage, total current, frequency and dissipated energy for the soft plasma jet operated with air and nitrogen gas are shown in Table 1. It can be seen that the values of breakdown voltage, total current and dissipated energy for the plasma jet operated with nitrogen gas are lower than that of (same) plasma jet operated with air gas.

**Table 1:** A comparison of the values of major plasma parameters for the soft plasma jet operated with air and nitrogen gas.

Parameters	Air gas	Nitrogen gas
Applied voltage (kV)	0.54	0.41
Total current (mA)	34.16	1.63
Frequency (kHz_)	~70	~70
Dissipated energy (mJ/s)	24.20	3.11

#### Optical characteristics

The optical emission spectra (OES) of the discharge calibrated in the units of absolute irradiance (µW/cm^2^/nm) recorded with HR4000 spectrometer (Ocean Optics Corporation) for plasma jet operated with air gas is shown in Fig. 1f. Emissions from various types of reactive oxygen and nitrogen species are clearly observed in the range of 200–900 nm. Between 200–300 nm, there are emissions from nitric oxide gamma (γ) band at various wavelengths of 236 nm, 246 nm, and 258 nm. These species are originated through the interaction of electrons as well as metastable atoms with oxygen and nitrogen molecules which are present in the air gas^18,19^. An emission from hydroxyl radical (OH^•^) of a very short lifetime is observed from 306–309 nm due to the interaction of energetic electrons/metastable atoms with water molecules present in the feeding gas as well as the ambient environment. OH^•^ radicals are strong precursors for the formation of long-lived reactive species like hydrogen peroxide (H_2_O_2_)^20^. There are also strong emissions from nitrogen second positive system (N_2_ SPS) from 315–380 nm and from nitrogen first negative system (N_2_ FNS) from 390–440 nm^18,19,21^. Excited nitrogen molecules not only combine with ambient oxygen molecules to form secondary reactive species but also they are able to dissociate water molecules and lead to the formation of OH^•^ radicals^22^. In addition to these species, there are also emissions from atomic oxygen (777 nm and 845 nm) and atomic nitrogen (747 nm and 868 nm). The emission for plasma jet operated with nitrogen gas is quite similar to that of air gas but the emission from atomic oxygen is absent (Fig. 1g).

### Temperature and pH estimation

MEM and RPMI cell culture serum-free media were placed in 48 well plates (1 ml per well) and exposed to air and N_2_ gas plasma for 30 sec, 60 sec, 180 sec, and 300 sec. After the exposure time, the temperature and pH of the media were measured in triplicate with an infrared (IR) camera (Fluke Ti100 Series Thermal Imaging Cameras, UK) and a pH meter (Eutech Instruments, Singapore).

### Cell lines for the study

The normal human dermal fibroblast (nHDF) and human melanoma cell lines (G-361, SK-MEL-31, and WM-266-4) were used to check the efficacy of air and N_2_ gas plasmas at different times and concentrations. All three melanoma cell lines were maintained in RPMI and MEM and supplemented with (10–15%) fetal bovine serum, 1% nonessential amino acids, 1% glutamine, 1% penicillin (100 IU/mL) and streptomycin (100 mg/mL) (Hyclone, USA) while nHDF cell line was maintain using FGM-2 bulletkit (Lonza, USA). All cultures were maintained at 37 °C, 95% relative humidity, and 5% CO_2_. The cells were grown in 24-well plate until confluence for experimentation.

### Alamar blue cytotoxicity assay

The cellular toxicity within all four cells was quantified with an Alamar Blue assay^23^, which is based on the enzymatic reduction of an indicator dye (resazurin to resorufin) by viable cells. Cells were seeded in 24 well plates for all experiments (80,000 cells/mL for G-361 cells and 50,000 cells/mL for nHDF, SK-MEL-31 and WM-266-4 cells). After 24 h of seeding, cells were given different doses of plasma from each gas. The distance between the nozzle of the jet plasma and upper surface of the cell culture media was kept at 1 cm throughout the experiments (Fig. 1b). The cells were incubated for 24 h after the plasma treatment. After 24 h, all of the media were drained off and replaced with fresh media containing Alamar Blue (diluted 1:10) and kept for 4 h until the color changed from blue to pink to indicate cell viability. The absorbance at 570 and 600 nm was measured with a microplate reader (Biotek, Vermont, USA). The cell viabilities were assessed as the ratio between the absorbance of the plasma-treated and normal cells^24,25^.

### Measurement of reactive species (H_2_O_2_ and NO_x_) in cell culture media

The levels of peroxide (H_2_O_2_) and different nitric oxide species (NO_2_ and NO_3_) within the cell culture media (RPMI and MEM) were evaluated to compare the generation of reactive species after giving plasma treatment (air and N_2_). The reactive species were measured after 30 sec, 60 sec, 180 sec and 300 sec.^26^ plasma exposure and estimated in micromoles per liter (μM) according to the manufacturer’s protocol (BioAssay System, CA, USA). The absorbance was calculated at 585 nm for the H_2_O_2_ and 540 nm for the nitric oxides (NO_x_) radicals.

### Apoptosis detection in human melanoma cells by DAPI staining and western blotting

All the three cells were cultured in their respective media overnight in glass bottom culture dish 35 mm^2^ (Nest, New Jersey, USA) before treatment with softjet plasma using air and N_2_ feeder gas. The cells were treated with 300 sec air CAP and 300 sec N_2_ CAP and kept it for 24 h in CO_2_ incubator. After incubation, the cells were fixed with 3.7% paraformaldehyde and washed 2X with sterile PBS. Afterwards, the cells were permeabilized with 0.5% Triton X-100 in PBS for 5 min and stained with DAPI (1 mg/ml) for 30 min at room temperature. The cells were again re-washed with PBS to avoid excessive staining and fluorescent images were captured and analysed using Olympus IX83-FP confocal microscope (Tokyo, Japan). For western blotting, cells were harvested after treated with CAP for 3 min and 5 min with air and N_2_ and then washed with PBS and lysed with RIPA buffer. Protein was quantified and separated on SDS-PAGE and transferred to nitrocellulose membrane. Primary antibodies for cleaved caspase 3 (anti-rabbit monoclonal, cell signaling Technology Inc., Danvers, MA, USA) and GAPDH (anti-mouse monoclonal, Bio-Rad, CA, USA) were incubated with membrane. The membrane was exposed with horseradish peroxidase (HRP)-conjugated secondary IgG antibodies.

### Regulation of gene expression analysis using q-PCR in human melanoma cells

Any alteration in the cellular level must be associated with changes in gene expression at the mRNA level. To clarify in molecular levels, we identified some genes that responsible for the cellular apoptosis and degradation of cell membranes by the production of plasma generated reactive species. The primers of apoptotic genes (*p53, bax, casp8*), mitogen-activated protein kinase (*map38*), and different types of NADPH oxidase genes (*nox1-5*) were designed and synthesized. All three melanoma cells were grown in 24 well plates and treated with softjet plasma using air and N_2_ gas separately. After incubation for 24 h, the cells were trypsinized and collected for RNA isolation using Trizol (Invitrogen, USA), after which q-PCR was performed with a Biorad 2X SYBR green mix. Reactions were carried out in a Biorad thermal cycler (Biorad, Korea), and the results were expressed as the fold change calculated with the ΔΔC_t_ method relative to a control sample^27^. Meanwhile, 18s rRNA was used as an internal normalization control. Quantitative real-time PCR was performed according to the forward and reverse primer sequences listed in Table 2.

**Table 2:** Primer sequence used for the evaluation of mRNA sequence; F: forward primer; R: reverse primer.

Name	Sequence (5′-3′)
*18s rna F*	CAGGTCTGTGATGCCCTTAGA
*18s rna R*	GCTTATGACCCGCACTTACTG
*p53 F*	GCCCCTCCTCAGCATCTTATC
*p53 R*	AAAGCTGTTCCGTCCCAGTAG
*bax F*	AAGAAGCTGAGCGAGTGTCTC
*bax R*	GCTGGCAAAGTAGAAAAGGGC
*casp8 F*	CCCAAATCAACAAGAGCCTGC
*casp8 R*	TCAGACAGTATCCCCGAGGTT
*map38 F*	CAGTCAGGTTCAGGTTGTGCT
*map38 R*	GGGCTTCTTTGTTAGGGTTTG
*nox1 F*	TCACCCCCTTTGCTTCTATCT
*nox1 R*	ATTCCTCCATCTCCTGTTCCA
*nox2 F*	TCAAGATGCGTGGAAACTACC
*nox2 R*	TTCAGATTGGTGGCGTTATTG
*nox3 F*	TATTGGCGTGTTCTTCTGTGG
*nox3 R*	TTCCTGGTGGAGTTCTTTGG
*nox4 F*	TCTGTTTGCTTGGGTTCATCT
*nox4 R*	AATCTCCTGGTTCTCCTGCTT
*nox5 F*	AGGCACCAGAAAAGAAAGCA
*nox5 R*	TCCATCTCCAGTTTAGTCAGCA

### Computational modeling

**Figure 2:**
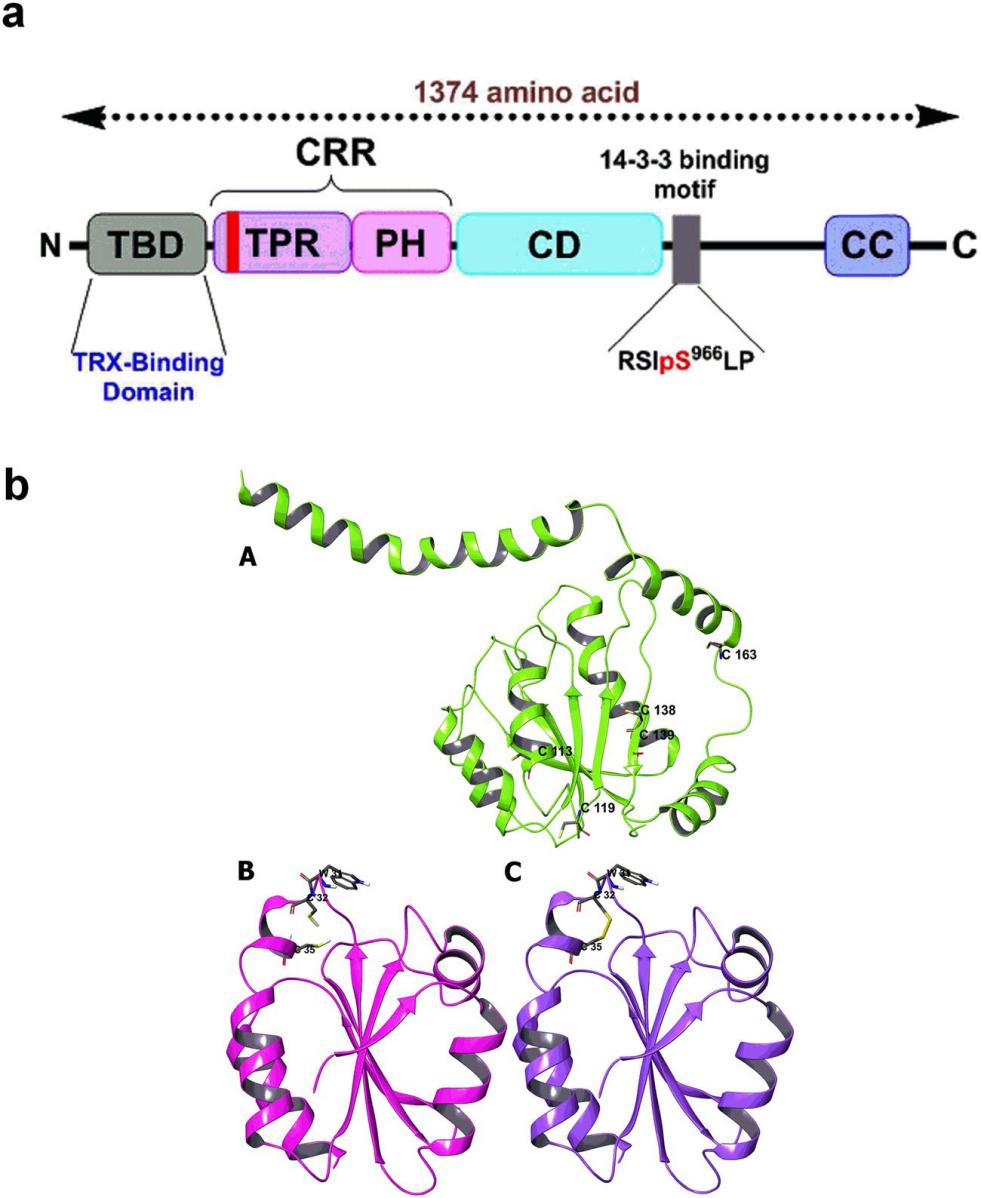
The schematic representation of the domain structure of ASK1. **(a)** TBD, TRX-binding domain; CRR, central regulatory region; TPR, tetratricopeptide repeats domain; PH, pleckstrin homology domain; CD, catalytic domain; CC, coiled-coil region. **(b)** (A) The ASK1-TBD (TRX-Binding domain) modeled protein with key amino acid residues, *i.e*., C98, C113, C119, C138, C139, and C163 (amino acid residues presented as single letter code); (B) The TRX-Reduced protein (PDB ID: 1ERT), and (C**)** The TRX-Oxidized protein (with key amino acid residues i.e. W31, C32, C35).

The role of peroxide (H_2_O_2_) and nitrogen (NO_x_) species in regulating the activity of the apoptotic pathway was determined by protein-protein docking and interaction analysis of apoptotic proteins: the apoptosis signal-regulating kinase 1 (ASK1) and thioredoxin 1 (TRX1) proteins. The human ASK1 protein has 1374 amino acids and consists of three domains: the N-terminal TRX-binding domain (ASK1-TBD), central regulatory region (ASK1-CRR) containing the TRAF-binding region, and serine/threonine kinase domain (ASK1-CD) located approximately in the center of the molecule^28,29^ **(**Fig. 2a**)**.

### Protein preparation

The three-dimensional structure of ASK1-TBD was still unknown and domain positioned between residues 46 and 277 in the N-terminal part of ASK1 **(**Fig. 2a**)**. The homology model of ASK1-TBD was resolved by Obsil *et al*.^29,30^, whereas reduced TRX^31^ protein was obtained (PDB ID: 1ERT) in our study. To model the TRX (oxidized) caused by reactive oxygen and nitrogen species (RONS), an intramolecular disulfide bond was created between the Cys32 and Cys35 residues **(**Fig. 2b**)**. These modeled protein structures were prepared according to the standard protocol: the addition of H-atoms, optimization of the side chain and restrained minimization until a root-mean-square (RMS) gradient of 0.3 Å is reached^32^.

### Protein-protein interaction

For the protein-protein interaction analysis, the homology model ASK1 was used as the receptor and TRX1-reduced and TRX1-oxidised were used as the ligand. Docking was performed with the FRODOCK 2.0 program^33,34^, which identified the interaction-energy minima through a fast and exhaustive rotational docking search combined with translational scanning. The binding energy was approximated with four types of potentials: van der Waals, electrostatics, desolvation, and new coarse-grained knowledge-based protein docking. The program produced 1000 poses, and the cluster and absolute energy score were calculated.

## Results

### Estimation of physical parameters (pH and temperature)

**Figure 3:**
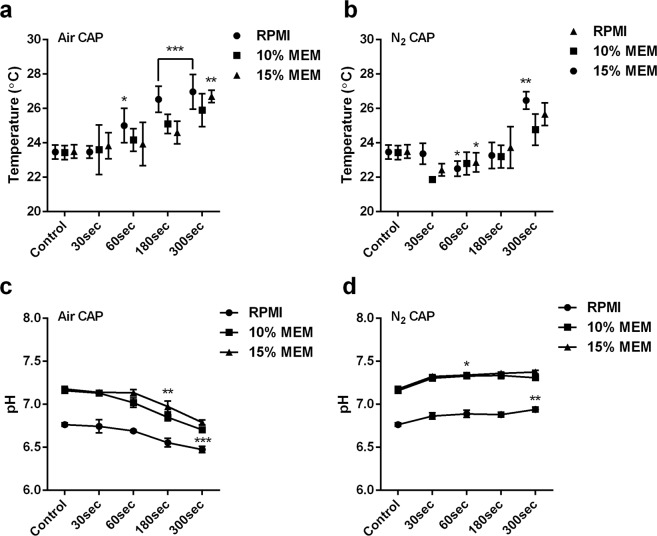
Effect of temperature and pH. **(a,b)** estimation of cell culture media temperature using air and N_2_ plasma and **(c,d)** estimation of pH using air and N_2_ plasma within cell culture media; after giving plasma treatment of 30 sec, 60 sec, 180 sec and 300 sec. Student’s t-tests were performed, and the levels of significance are indicated as follows: *p < 0.05; **p < 0.01; and ***p < 0.001.

Previous studies using CAP with various jet plasma devices demonstrated that physical parameters within the solutions were changed, such as an increase in temperature^26^. However, softjet CAP device (PBRC, Seoul) was used in the present study was modified to overcome the heating effect which was common issues in previously used plasma devices. With air plasma, the temperature in cell media (RPMI, 10% and 15% MEM) showed an increase in temperature after 60 sec, 180 sec, and 300 sec treatment time (Fig. 3a). However, with N_2_ plasma, the temperature slightly dropped after 60 sec treatment which later marginally increased and reached a maximum of up to 26.4 °C in RPMI media after 300 sec of treatment (Fig. 3b). This mainly attributed because of the softjet device built to minimize the heating effect at the treatment time.

The pH is dependent on primary free radicals generated by CAP and secondary free radicals produced by interaction with cell media and hence estimated for 30 sec, 60 sec, 180 sec, and 300 sec. Using air plasma, in the RPMI media, the measured pH slightly decreased after 30 sec (6.64) which declined further to 180 s (6.55) and 300 s (6.47) as compared to control (6.7). The MEM media (10% and 15%) showed slightly decreased in pH after 30 sec, however, 60 sec, 180 sec, and 300 sec showed noticeable pH decreased. MEM 10% decreased pH 7.02, 6.85, and 6.7 while, for 15% MEM, the pH also decreased in a time-dependent manner but was higher at 60 sec (7.13), 180 sec (6.97), and 300 sec (6.79) (Fig. 3c).

However, N_2_ plasma exhibited a contrasting effect on the three different cell media compared to air plasma. Overall, it showed a slightly increased in pH at 30 sec treatment which was remained constant at later treatment time **(**Fig. 3d**)**.

### Effect of air and N_2_ CAP on H_2_O_2_ and NO_x_ levels within cell media

**Figure 4:**
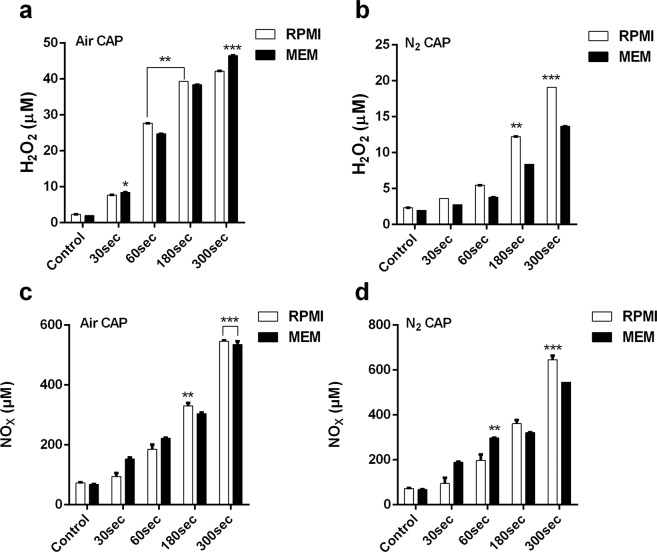
Free radical after giving softjet plasma treatment within cell media**. (a,b)** H_2_O_2_ level estimation using air and N_2_ cold plasma; **(c,d)** nitric oxide level estimation using air and N_2_ cold plasma. Student’s t-tests were performed, and the levels of significance are indicated as follows: *p < 0.05; **p < 0.01; and ***p < 0.001.

With feeder gases, softjet CAP can generate a different kind of free radicals, ions, and UV rays that reacts with the liquid cell culture medium to form secondary reactive stable species like H_2_O_2_, NO_2_, and NO_3,_ etc. Using air CAP the level of H_2_O_2_ within both cell media (RPMI and MEM) increased significantly at all treatment times as compared to the control. The softjet air CAP for 60 sec induced H_2_O_2_ generation (~25 μM) within the RPMI media, which increased at 180 sec (40 μM) and 300 sec (42 μM) of treatment. The MEM media showed comparable H_2_O_2_ levels at 30 sec and 60 sec treatment while it was higher after 300 sec (46 μM) of treatment (Fig. 4a) as compared to RPMI media. With N_2_ plasma the H_2_O_2_ level in the RPMI and MEM media slightly increased at 30 sec and 60 sec treatment. However, H_2_O_2_ level alleviates significantly at 180 sec (12 μM) which reaches a maximum at 300 sec (20 μM) treatment for RPMI media. In contrast, the MEM media also exhibit an increase in the H_2_O_2_ level at 180 sec (7 μM) and 300 sec (14 μM) respectively (Fig. 4b).

The level of NO_x_ (NO_2_ and NO_3_) radicals within RPMI cell media, increased after air plasma treatment at all-time intervals, but MEM showed a higher NO concentration (152.9 μM) than RPMI (94 μM) after 30 sec and 60 sec of treatment and reached its maximum at 300 sec (545 μM) (Fig. 4c).

N_2_ plasma revealed more intense NO_x_ production than the air plasma in both cell culture media. The NO_x_ concentration increased significantly (p < 0.001) in the RPMI media after 30 sec treatment compared to the control and it revealed 60 sec (197 μM), 180 sec (361 μM), and 300 sec (646 μM) NO_x_ species. However, MEM media showed a higher NO_x_ concentration with N_2_ plasma treatment at 30 sec (188 μM) and 60 sec (298 μM) compared to the RPMI media at the same time intervals. Though, NO_x_ concentration increased only slightly at 180 sec (321 μM) and reached a maximum at 300 sec (545 μM) (Fig. 4d).

### Estimation of CAP-induced toxicity

**Figure 5:**
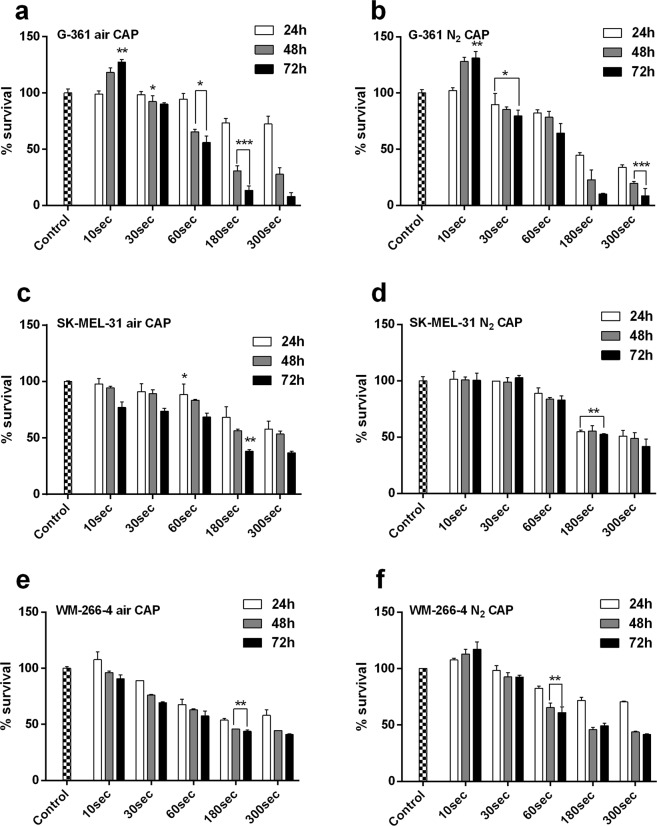
Assessment of % survival within different human melanoma cell lines at different plasma doses of 10 sec, 60 sec, 180 sec and 300 sec using softjet plasma (air and N_2_ gas) at 24 h, 48 h, and 72 h incubation time-intervals. **(a,b)** G-361 human melanoma cells; **(c,d)** SK-MEL-31 human melanoma cells and **(e,f)** WM-266-4 human melanoma cells. Student’s t-tests were performed, and the levels of significance are indicated as follows: *p < 0.05; **p < 0.01; and ***p < 0.001.

Cellular toxicity induced by softjet air and N_2_ CAP in three human melanoma cell lines (G-361, SK-MEL-31, and WM-266-4) were estimated using Alamar Blue^Tm^ cytotoxicity assay at 10 sec, 30 sec, 60 sec, 180 sec, and 300 sec. G-361 cells showed proliferation at 10 sec treatment at all-time intervals. The cytotoxicity increased at 180 sec of air CAP treatment (70% survival) and remained the same after 300 sec treatment when incubating for 24 h. G-361 cells showed higher toxicity when receiving air and N_2_ CAP for 180 sec and 300 sec when incubating for 48 h and 72 h. CAP treatment using air for 60 sec declined survival rate (58%), which increased further after 180 sec (18% survival) and 300 sec (7% survival) of treatment (Fig. 5a). N_2_ CAP also showed similar trend like air CAP. The cytotoxic effect was higher after 60 sec at 72 h incubation (45% toxicity) and reached a minimum % survival at 300 sec at all time-intervals (Fig. 5b). For SK-MEL-31, softjet air CAP didn’t show much cytotoxicity at 10 sec and 30 sec treatment time at 24 h and 48 h. However, SK-MEL-31 showed much higher cytotoxicity (60%) when incubated for 48 h and 72 h at 180 sec and 300 sec. The N_2_ CAP also showed similar effects like air CAP (Fig. 5c) after giving 10 sec and 30 sec treatments. However, at 60 sec treatment time, the % survival declined at all three time-intervals. At 180 sec treatment, the cytotoxicity level increased significantly (p < 0.001) as compared to control and remained constant at 300 sec treatment time (Fig. 5d). Softjet air plasma treatment on WM-266-4 initially showed very low toxicity as compared to control and comparable. Though % survival at 180 sec CAP treatment, cytotoxicity was much higher and it remains the same at 300 sec (Fig. 5e) which keeps on decreasing at all CAP doses and at all time-intervals.

For WM-266-4, N_2_ CAP treatment decrease % survival with 60 sec CAP treatment and continued to decrease with longer CAP treatment times up to 300 sec for 48 h and 72 h incubation periods **(**Fig. 5f**)**. However, nHDF cells showed non-significant effects while receiving air and N_2_ plasma jet at all time-intervals **(**Fig. S1**)**. The microscopic data by differential interference contrast (DIC) images revealed that air CAP reduces the number of cells with higher pace as compared to N_2_ CAP after receiving CAP for 300 sec **(**Fig. S2**)**. Based on the data from the soft jet plasma using air and N_2_ for the three melanoma cell lines, we received IC_50_ of all three cell lines at 180 sec (3 min) and 300 sec (5 min) and hence kept these plasma doses constant for the study.

### Apoptosis detection by DAPI staining, western blotting and estimation of apoptotic and oxidative stress-related genes by air and N_2_ CAP treatment

**Figure 6:**
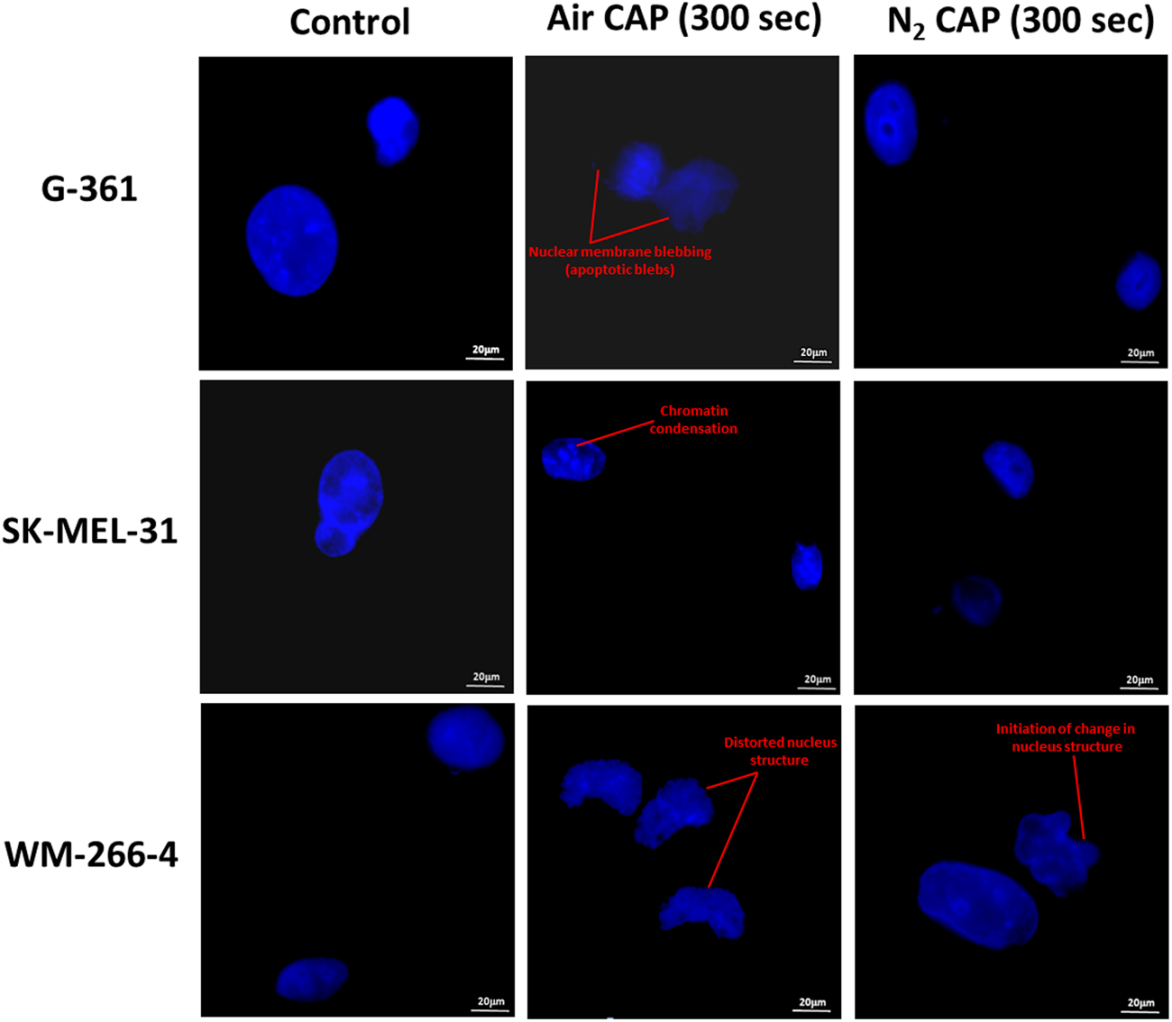
Detection of apoptosis by DAPI staining nuclei of G-361, SK-MEL-31 and WM-266-4 melanoma cells treated by using air and N_2_ CAP at 300 sec. (Magnification = 100X).

The result outcome by individual treatment of 300 sec Air CAP significantly provoked apoptosis as compared to 300 sec N_2_ CAP treatment. The induction of apoptosis was evident by DAPI staining using 100X magnification (Olympus IX83-FP confocal microscope, Tokyo, Japan) from the initial changes in the structure of nucleus (shrinkage) alonwith the occurrence of apoptotic bodies, nuclear membrane blebbing and chromatin condensation and fragmentation^35^ as shown in Fig. 6.

However, 300 sec N_2_ CAP didn’t affect much on nucleus structure except some initial changes in nuclear membrane, while control group shows prominent round shaped nucleus in all 3 human melanoma cell lines.

**Figure 7:**
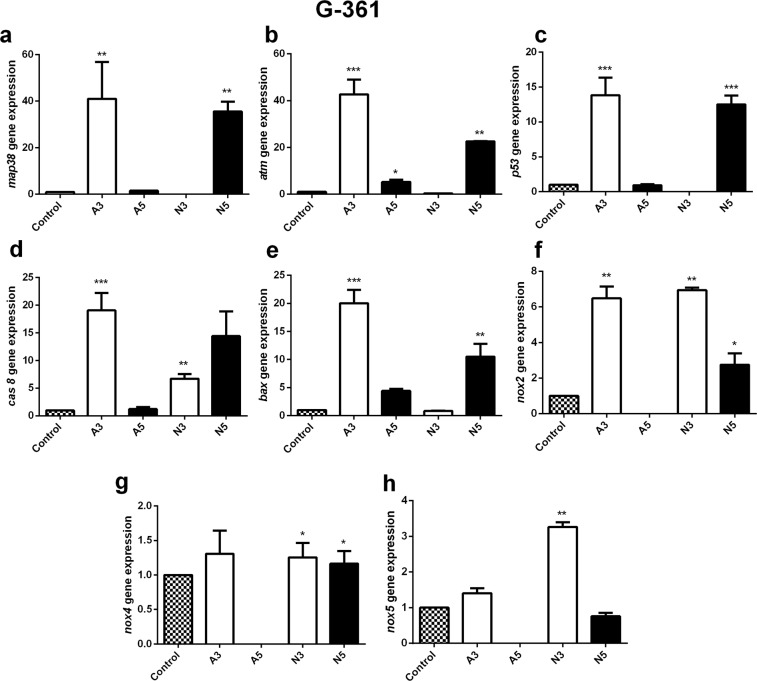
The relative value of mRNA expression of apoptosis and oxidative stress-related genes in G-361 human melanoma cells after plasma exposure for 180 sec (A3/N3) and 300 sec (A5/N5) with air and N_2_ cold plasma. The relative mRNA expression of *map38*, atm, *p53*, *cas8*, *bax* and *nox(1–5)* subfamily were measured by real-time RT-PCR and *18s rRNA* was used as a reference gene. Student’s t-tests were performed, and the levels of significance are indicated as follows: *p < 0.05; **p < 0.01; and ***p < 0.001.

The present study illustrated that ROS induction after treatment with air and N_2_ CAP increased the levels of intracellular ROS that may be mediated through *nox-1* and *p53/atm* gene expression levels. The generation of ROS by nicotinamide adenine dinucleotide phosphate-oxidase (*nox*) in response to apoptosis-related signaling is essential for signal transduction^36,37^. *atm* belongs to one of the largest protein kinase families and is responsible for regulating cellular responses and activating H_2_AX and *p53* in the DNA damage signaling cascade^38^. G-361 cells showed an increase of all apoptotic genes and *nox* 2, 4, 5 genes by A3 plasma treatment **(**Fig. 7f–h**)**. The level of *map38* and atm genes increased up to 40 folds **(**Fig. 7a,b**)** while *p53* increased 14 folds **(**Fig. 7c**)** as compared to control. The level of *cas8* and *bax* also increased 20 folds after A3 treatment **(**Fig. 7d,e**)**. While at A5 treatments all the apoptotic genes decreased significantly as compared to A3 and comparable to the control group.

N3 treatment in G-361 cells has no effects on *map38*, atm, p53, and bax but cas8 was increased (Fig. 7a–e). All apoptotic genes were increased at N5 treatment results in an increase in apoptotic genes. The *nox* 1 and 3 gene expressions were not detected for the G-361 cell line, while *nox 2* and *nox* 4 increased after A3 and N3 treatments.

**Figure 8:**
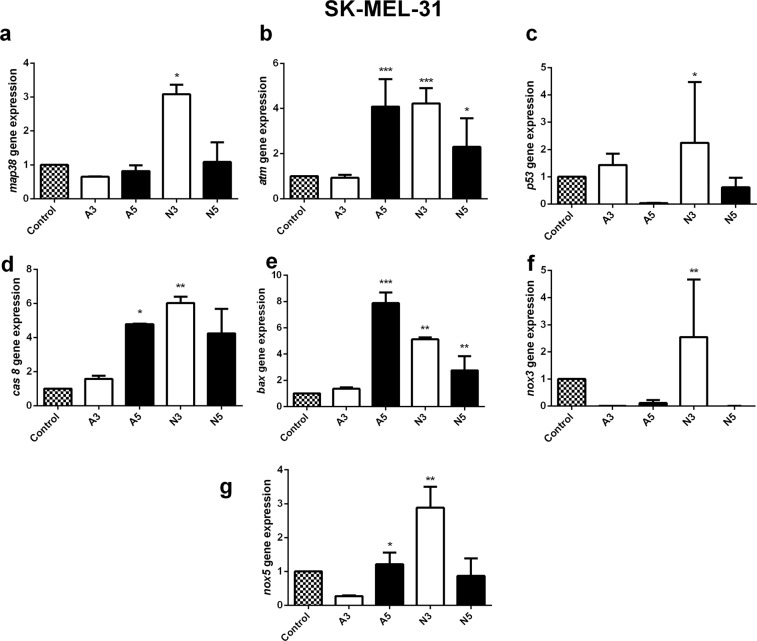
The relative value of mRNA expression of apoptosis and oxidative stress-related genes in SK-MEL-31 human melanoma cells after plasma exposure for 180 sec (A3/N3) and 300 sec (A5/N5) with air and N_2_ cold plasma. The relative mRNA expression of *map38*, atm, *p53,* *cas8,* *bax* and *nox(1–5)* subfamily were measured by real-time RT-PCR and *18s rRNA* was used as a reference gene. Student’s t-tests were performed, and the levels of significance are indicated as follows: *p < 0.05; **p < 0.01; and ***p < 0.001.

SK-MEL-31 melanoma cells revealed an increase in *p53, cas8,* and *bax* levels after CAP treatment for A3, A5, and N3. The expression of apoptotic genes after N_2_ CAP treatment N3 significantly increased in all groups (Fig. 8). *nox* *1, 2,* and *4* were not detected in the SK-MEL-31 cell line. However, an increased level of *nox 3* was only detected N3 CAP treatment (3-fold), and the other treatments only showed insignificant increases (Fig. 8f). An increased level of *nox* *5* was detected after A3 (3-fold) and A5 (2.5-fold) treatment, while the level of *nox* *5* decreased after N_2_ CAP treatment for N5 (4.5-fold) compared to N3 (7-fold) respectively (Fig. 8g).

**Figure 9:**
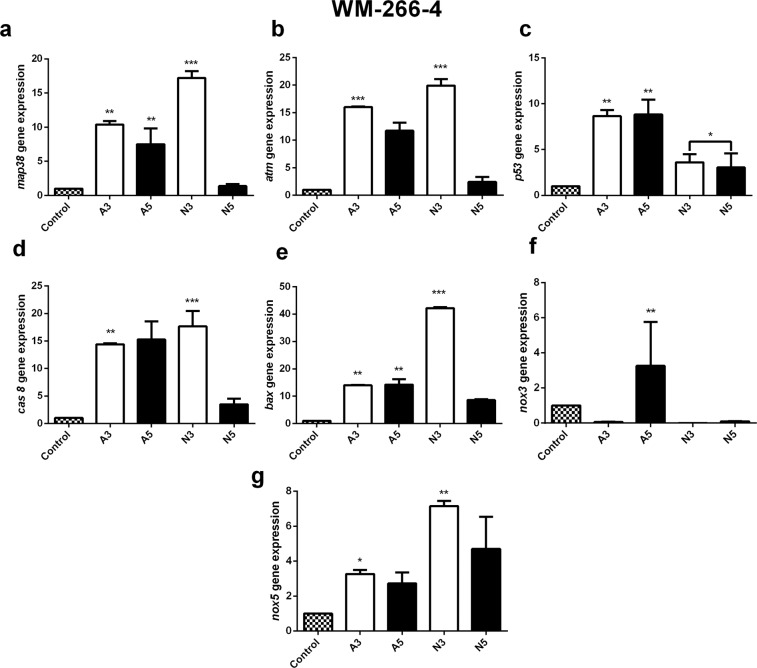
The relative value of mRNA expression of apoptosis and oxidative stress-related genes in WM-266-4 human melanoma cells after plasma exposure for 180 sec (A3/N3) and 300 sec (A5/N5) with air and N_2_ cold plasma. The relative mRNA expression of *map38*, *atm, p53,* *cas8,* *bax* and *nox(1–5)* subfamily were measured by real-time RT-PCR and *18s rRNA*was used as a reference gene. Student’s t-tests were performed, and the levels of significance are indicated as follows: *p < 0.05; **p < 0.01; and ***p < 0.001.

WM-266-4 melanoma cells showed an increase in all apoptotic genes after treating with air plasma (A3 and A5). Gene expression for *map38* (10-folds), *atm* (15-folds), *p53* (8-folds), *cas8* (14-folds) and *bax* (15-folds) was increased after receiving A3 and comparable to *p53, cas8* and *bax* after receiving A5 (Fig. 9a–e).

N_2_ CAP treatment (N3) increased some apoptotic gene expressions level. It increased *map38* (18-folds), *atm* (18-folds), *cas8*(17-folds) and *bax* (40-folds) expression respectively compared to the control cells. N5 treatment didn’t show much changes in the expression level of *map38* and *atm* gene and compared to control. N5 treatment also increased the level of *p53* (3-folds), *cas8* (2-times) and *bax* (8-times) (Fig. 9a–e). *nox3* gene was only expressed in A5 group while *nox5* gene expression was increased in A3 and A5 (Fig. 9f,g).

Using western blot analysis we accessed the stimulation of caspase 3 using both air and nitrogen CAP treatment on SK-MEL-31 and WM-266-4 human melanoma cell lines. *cas3* activation was found in both of treatment group used with air and nitrogen gas and it shown the *cas3* expression significantly increased in 5 min air CAP as compared to N_2_ CAP. It indicate parallel result with q-PCR gene expression especially with *cas8* expression (Fig. S4).

## Discussion

The poor diagnosis of metastatic melanoma despite conventional therapies has led to the development of new therapeutic strategies that not only treat the disease but also minimize the post-treatment effects^39^. Advances in model systems that closely simulate the human situation are much needed^40^. In the present study, we used a softjet CAP device with air and N_2_ feeder gases to analyze the inhibition rate in three human melanoma cell lines at different time intervals. The results revealed that the softjet CAP induced high cell death in the G-361, SK-MEL-31, and WM-266-4 human melanoma cells.

The physical characterization of softjet plasma devices by using both gases (air and N_2_) revealed the production of highly reactive radicals. CAP originating using the ambient air contained radicals mainly from N_2_ and O_2_ (*e.g*., N, O, O_3_, NO, NO_2_, OH^•^), while the CAP using N_2_ gas contained radicals of only unstable nitrogen species. The final products of these unstable species that reacted with the cell culture media results in the production of H_2_O_2_ and NO_x_, which are quite stable. Even though it is still unclear which component of the softjet plasma induces toxicity. Therefore, we presume that this dose-dependent toxicity has the same tendency as the amounts of dissolved H_2_O_2_ and NO_x_ in solution in published reports^41^. G-361 and SK-MEL-31 exhibited maximum cytotoxicity with 180 sec of air CAP treatment after 48 h and 72 h of incubation, respectively. This may be because of the interaction of CAP with liquid, which leads to various kinds of ROS in the culture media and linearly increases the level of intracellular ROS^42,43^. Because of the dependency of endogenous ROS generation on chemical induction, this direct ROS generation can be a good strategy for reducing melanoma. Previous reports stated that plasma induces cell death and reduced cell adhesion while interacting with organic components in cell culture media^27,44^, which leads to toxicity at later time intervals.

The cell death induced by soft jet plasma using 300 sec air and N_2_ CAP showed apoptosis in the G-361, SK-MEL-31 and WM-266-4 melanoma cells which was apparent by the DAPI staining method. This can plausibly be explained by following two main processes: extrinsic and intrinsic pathways. Signalling from the death receptors plays a key role in mediating external signals to caspase8 in the extrinsic pathway. Also, CAP treatment generate O_2_^•−^ which on reaction with liquid cell culture medium may produce HO_2_^•^ which is a strong oxidant than O_2_^•−^ and it can initiate lipid peroxidation of melanoma cells. NO_2_^−^ is more unstable than NO_3_^−^ and undergoes very fast decomposition which leads to NO_2_^•^ and OH^•^. These free radicals generation lead to another reason for apoptosis in melanoma.

In the molecular level, q-PCR analysis of the G-361 cells and WM-266-4 cells also revealed an increase in the *casp8* signal after 180 sec (A3) of air CAP treatment and 300 sec (N3) of N_2_ plasma treatment and this leads to the apoptosis. *bax* is a *bcl-2* family gene that is related to the signaling of early apoptosis^45^. It was also reduced with A3 plasma treatment and N3 plasma treatment in the G-361 cells and A3 plasma treatment and N3 plasma treatment in the WM-2664 cells. However, SK-MEL-31 showed more necrosis compared to the other two melanoma cell lines. Plasma treatment of A3 showed 22.41% necrosis, while A5 treatment decreased the necrosis to 5.5%. This was revealed by the *casp8* and *bax* activity at the same given CAP doses. Apart from the oxidative stress induced by RONS generated from air and N_2_ CAP, *map38* was generally phosphorylated^46^. Upregulation of phospho *map38* signaling after A3 plasma treatment of G-361 and SK-MEL-31 and N3 CAP treatment of SK-MEL-31 and WM-2664 may upregulate apoptotic genes through distinct phosphorylation events. This can result in stress stimulating DNA damage^46^ and hence tumor cell death.

In our study, the softjet CAP using air and N_2_ ambient gases produced oxidative potential that was partially mediated through *nox* *1–5* enzymes and *p53/atm* overexpression. The gene overexpression in all three melanoma cell lines clearly indicates that RONS-dependent DNA damage triggered signaling leading to cell death. However, the actual damage to the different melanoma cells and the underlying mechanism must be studied to minimize the unexpected side effects of plasma medicine.

**Figure 10:**
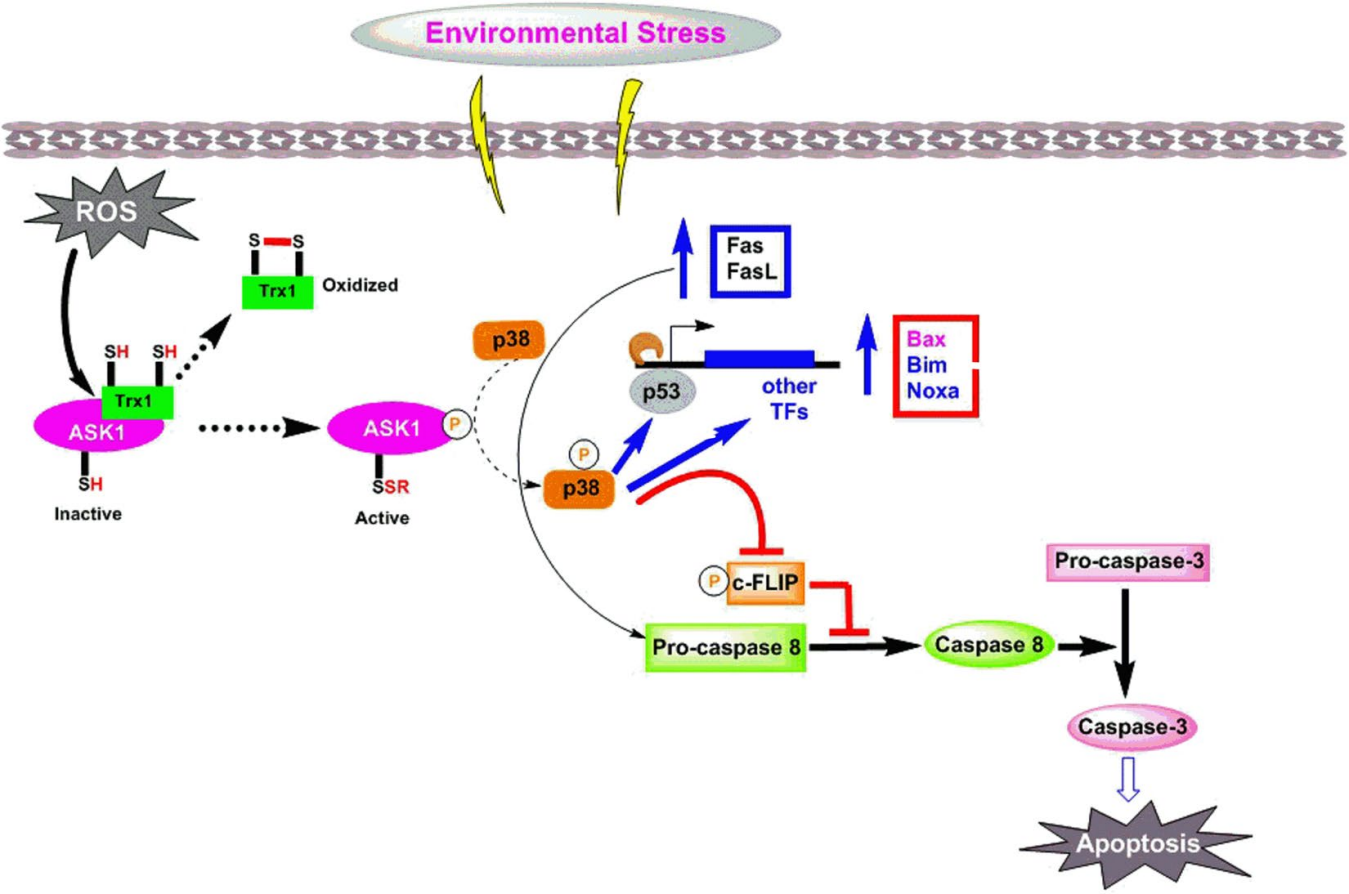
Illustrating the Reactive Oxygen and Nitrogen Species induced expression of the pro-apoptotic protein signaling cascade that leads to apoptosis.

Based on the results of our experimental and computational study, we also propose the following mechanism for the ROS-regulated activation of *p38* pathways and subsequent signaling events that lead to apoptosis (Fig. 10). The activation of the primary *p38* pathway has been reported to lead to apoptosis and is induced by the activation of ASK1^47,48^. In contrast, oxidative stress induced by RONS causes TRX to dissociate from ASK1, and the free ASK1 further activates *p38* pathways that lead to apoptosis^49,50^. The protein-protein interaction results for ASK1 and the TRX1-reduced form clearly showed that the catalytic residues Phe31, Cys32 and Cys35 of TRX1 oriented towards the Cys113 and Cys119 binding residues of ASK1. The calculated absolute energy score for this complex was 138,562.30 kcal/mol. Similarly, the analysis of the complex of ASK1 and the TRX1-oxidised form showed a similar interaction with a decreased absolute energy score of 128,488.17 kcal/mol (Fig. S3). Our results corroborate earlier experimental studies showing that the activity of ASK1 is downregulated by the reduced TRX1 protein and upregulated when the TRX1 protein is oxidized (i.e. disulfide bond Cys32–Cys35). In other words, ASK1 is in its free form to propagate *p38*-mediated apoptosis^48,51–59^.

Oxidative stress with an increased concentration of RONS targets the ASK1:TRX1 protein complex and causes the release of TRX1 from ASK1 through the redox mechanism. The free ASK1 then phosphorylates p38 and activates the signaling cascade. The activated *p38* induces the expression of pro-apoptotic proteins such as Fas and its ligand (FasL), *bax*, *bim*, or *nox-a* (all from the *bcl-2* family) through the phosphorylation of the transcription factor *(p53)*. Then, *p38* can inhibit c-FLIP phosphorylation and impair its function as an inhibitor of *cas* *8* activations. The expressed pro-apoptotic proteins Fas and FasL activate pro-*cas8* to *cas8*. This further activates *cas3*, which contributes to apoptosis.

In conclusion, the exact mechanism for the inhibitory effect of CAP remains unknown and needs to be further investigated. However, our results showed that air and N_2_ softjet CAPs are useful and powerful agents against human melanoma *in vitro*, where the main effects seem to be the activation of apoptosis and an increase in metabolic toxicity. For therapeutic applications, the different cellular responses to plasma treatment should be further screened according to cell morphology and cancer genotype and must be compared with the normal cells. However, our preliminary results show potential for the preferential killing of melanoma cells with softjet air and N_2_ CAP treatment in melanoma therapy.

## Supplementary information

Supplementary Figures
