# Supplementary material for: Cold atmospheric plasma generated reactive species aided inhibitory effects on human melanoma cells: an in vitro and in silico study
Source: Sci Rep. 2020 Feb 25;10:3396. doi: 10.1038/s41598-020-60356-0 (PMC7042335; doi:10.1038/s41598-020-60356-0)
Supplement: Supplementary file 1 — Supplementary Figures [file 41598_2020_60356_MOESM1_ESM.pdf]

Supplementary Materials

Cold atmospheric plasma generated reactive species aided inhibitory effects on human melanoma cells: an *in vitro* and *in silico* study

Dharmendra Kumar Yadav[1,#*], Manish Adhikari[2,#], Surendra Kumar[1], Bhagirath Ghimire[2], Ihn Han[2], Mi-Hyun Kim[1], Eun-Ha Choi[2*]

[1]Gachon Institute of Pharmaceutical Science & Department of Pharmacy, College of Pharmacy,
Gachon University, Incheon, Republic of Korea

[2]Plasma Bioscience Research Center, Applied Plasma Medicine Center, Department of Electrical & Biological Physics, Kwangwoon University, Seoul, Republic of Korea

Email: dharmendra30oct@gmail.com; ehchoi@kw.ac.kr

*Corresponding author

Prof. Dharmendra Kumar Yadav, Ph.D
Assistant Professor
Office: +82-32-820-4947
Email: *dharmendra30oct@gmail.com*

Prof. Eun-Ha Choi, Ph.D
Professor & Director,
Office: +82-2-940-5236
Email: *ehchoi@kw.ac.kr*

[#]These authors contributed equally to this work and joint first authors.

Fig. S1 Assessment of % survival in normal human dermal fibroblast (nHDF) at different plasma doses of 10 sec, 30 sec, 60 sec, 180 sec and 300 sec using softjet plasma at 24 h, 48 h and 72 h incubation time-intervals using (a) air and (b) N_2 gas.

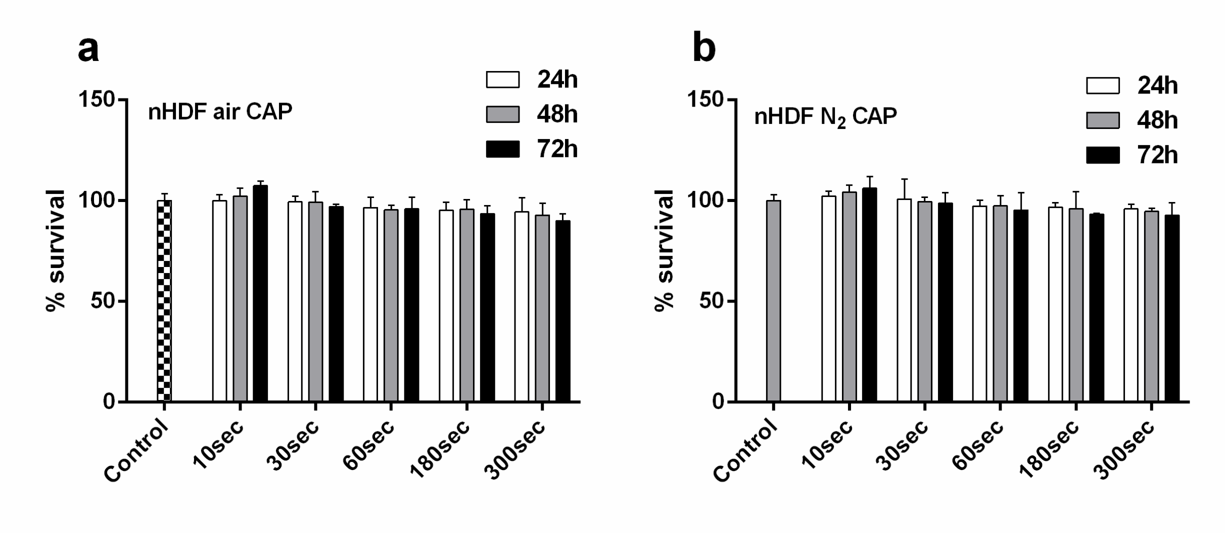

Fig. S2 Differential interference contrast (DIC) microscopy of nHDF, SK-MEL-31 and WM-266-4 cells by using air and N_2 CAP at 300 sec. (Magnification =10X)

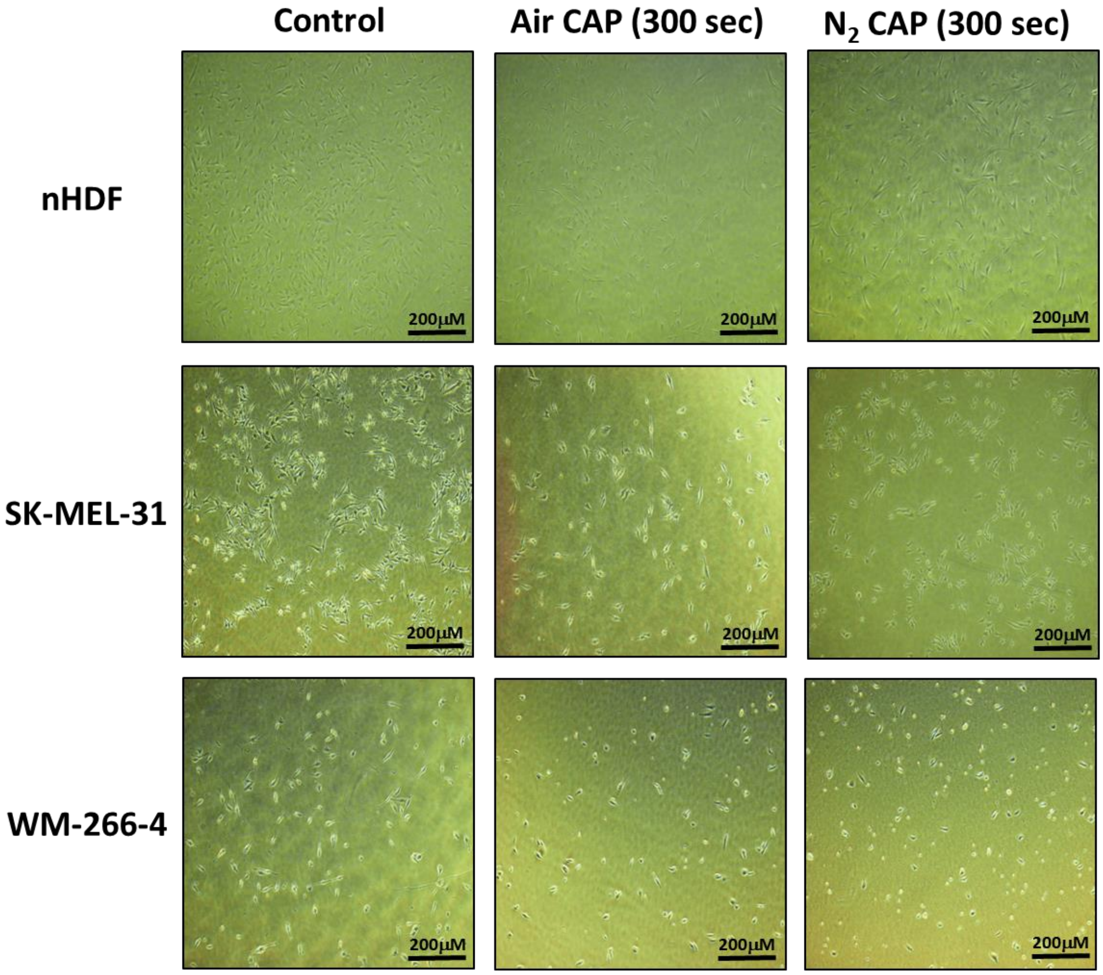

Fig. S3 Binding mode and interaction in reduced **(a)** and oxidized state **(b)**. **(a)** (A) ASK1 and TRX1-Reduced; (B) The catalytic motif of TRX1-Reduced juxtaposed in binding site of ASK1 rich in cysteine amino acid residues with absolute energy score of 138562.30 kcal/mol; **(b)** (A) ASK1 and TRX1-Oxidized; (B) The catalytic motif of TRX1-Oxidized juxtaposed in binding site of ASK1 rich in cysteine amino acid residues with absolute energy score of 128488.17kcal/mol.

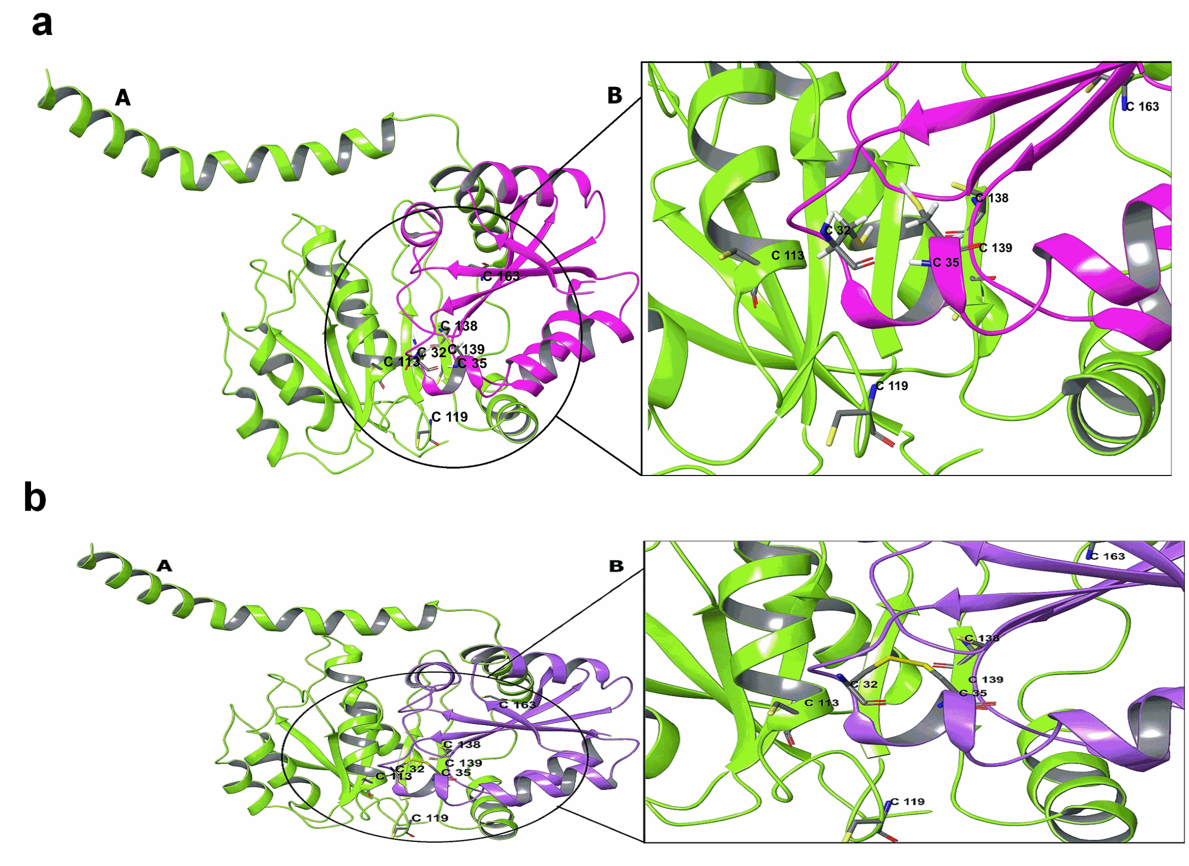

Fig. S4. Western blot analysis of cleaved caspase 3 on SK-MEL-31 and WM-266-4 human melanoma cell lines using 3min and 5min air and N_2 softjet CAP treatment. Total proteins were isolated from both cell lines after treatment. Each protein name mentioned corresponding to its protein band and GAPDH was taken as reference protein. The protein levels were detected using following antibodies: cleaved caspase (molecular weight 17-19kDa) and anti-GAPDH (molecular weight 36kDa). The full length gel images placed after the cropped images of the protein bands.

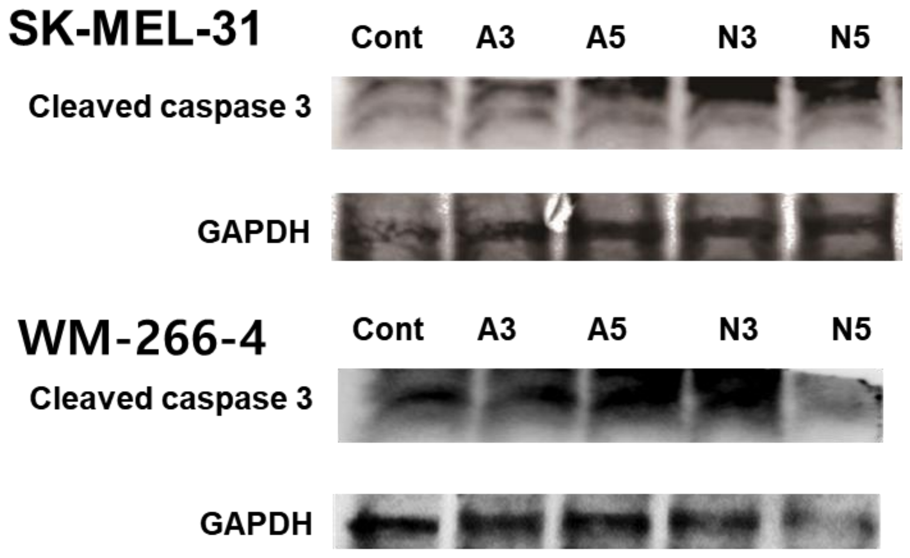

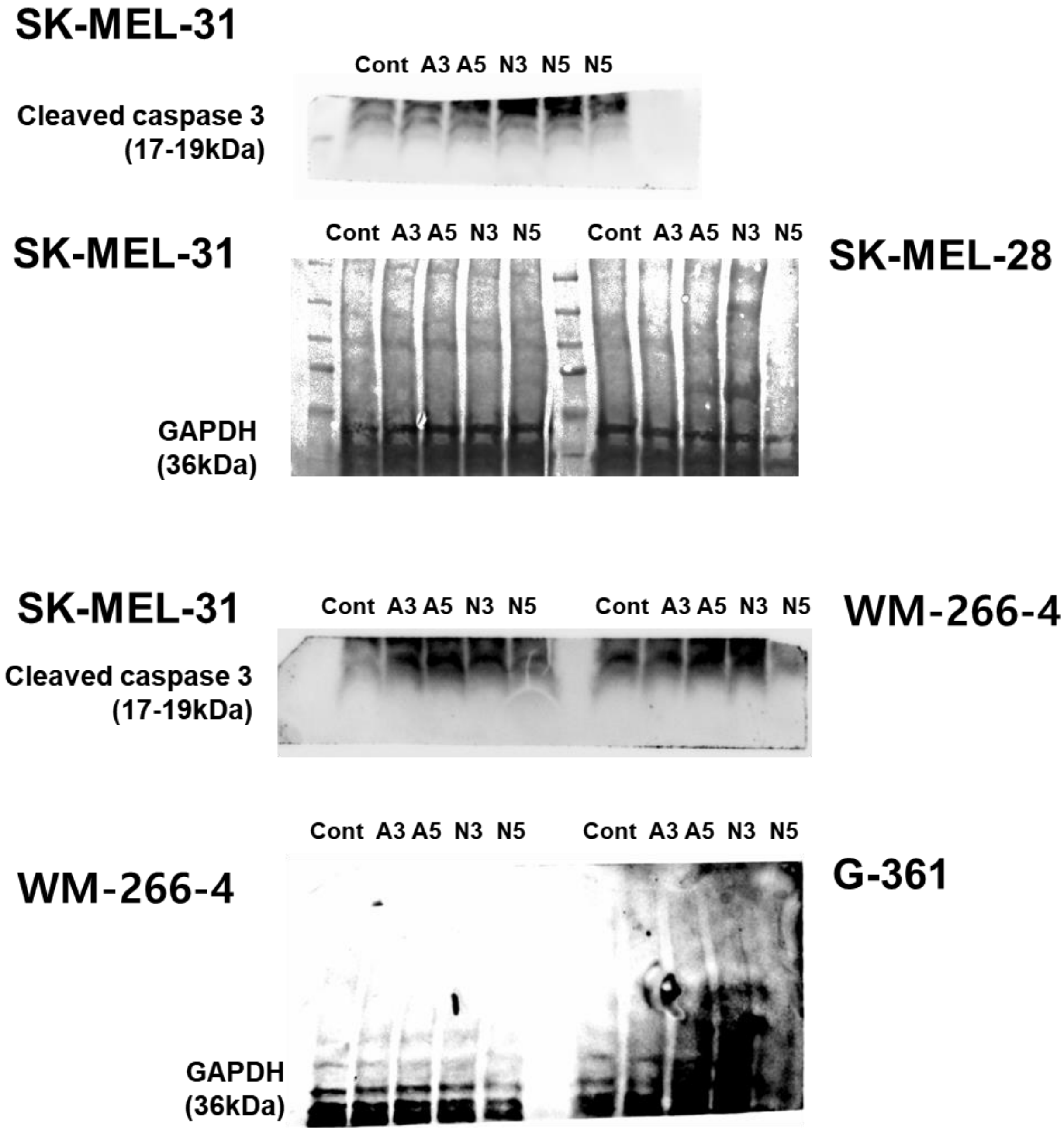
SK-MEL-31
Cont A3 A5 N3 N5 N5
Cleaved caspase 3
(17-19kDa)
SK-MEL-31
Cont A3 A5 N3 N5 Cont A3 A5 N3 N5
SK-MEL-28
GAPDH
(36kDa)
SK-MEL-31
Cont A3 A5 N3 N5 Cont A3 A5 N3 N5
WM-266-4
Cleaved caspase 3
(17-19kDa)
Cont A3 A5 N3 N5 Cont A3 A5 N3 N5
WM-266-4
G-361
GAPDH
(36kDa)
